# Significant elevation of biomarkers of myocardial necrosis after coronary artery bypass grafting without myocardial infarction established assessed by cardiac magnetic resonance

**DOI:** 10.1097/MD.0000000000006053

**Published:** 2017-02-10

**Authors:** Leandro Menezes Alves da Costa, Whady Hueb, Cesar Higa Nomura, Alexandre Ciappina Hueb, Alexandre Volney Villa, Fernando Teiichi Costa Oikawa, Rodrigo Morel Vieira de Melo, Paulo Cury Rezende, Carlos Alexandre Wainrober Segre, Cibele Larrosa Garzillo, Eduardo Gomes Lima, Jose Antonio Franchini Ramires, Roberto Kalil Filho

**Affiliations:** Department of Atherosclerosis, Heart Institute (InCor), University of São Paulo Medical School, São Paulo, SP, Brazil.

**Keywords:** biomarkers, coronary artery bypass grafting, coronary artery disease, myocardial infarction, troponin

## Abstract

The release of myocardial necrosis biomarkers after off-pump coronary artery bypass grafting (OPCAB) frequently occurs. However, the correlation between biomarker release and the diagnosis of procedure-related myocardial infarction (MI) (type 5) has been controversial. This study aimed to evaluate the amount and pattern of cardiac biomarker release after elective OPCAB in patients without evidence of a new MI on cardiac magnetic resonance (CMR) imaging with late gadolinium enhancement (LGE).

Patients with normal baseline cardiac biomarkers referred for elective OPCAB were prospectively included. CMR with LGE was performed in all patients before and after interventions. Measurements of troponin I (cTnI) and creatine kinase MB fraction (CK-MB) were systematically performed before and after the procedure. Patients with new LGE on the postprocedure CMR were excluded.

All of the 53 patients without CMR evidence of a procedure-related MI after OPCAB exhibited a cTnI elevation peak above the 99th percentile. In 48 (91%), the peak value was >10 times this threshold. However, 41 (77%) had a CK-MB peak above the limit of the 99th percentile, and this peak was >10 times the 99th percentile in only 7 patients (13%). The median peak release of cTnI was 0.290 (0.8–3.7) ng/mL, which is 50-fold higher than the 99th percentile.

In contrast with CK-MB, considerable cTnI release often occurs after an elective OPCAB procedure, despite the absence of new LGE on CMR.

## 1 Introduction

The elevation in biomarkers of myocardial necrosis is frequent after cardiac revascularization procedures. The pathogenic process underlying this phenomenon is complex and is still not well understood. It has been the subject of many studies and has been defined in various ways. Many factors may be responsible for this elevation, and even the manipulation of the heart that is required during mechanical revascularization procedures may predispose to myocardial injury. Several definitions have been suggested for the diagnosis of myocardial infarction (MI) after coronary artery bypass grafting (CABG), and in recent years the definitions have been updated due to limitations found in the previous reports. The elevation of cardiac biomarkers is considered the main event for the diagnosis, although elevated biomarkers are not conclusive evidence of cardiac injury. The current universal definition suggested by the European Society of Cardiology (ESC)/American College of Cardiology Foundation (ACCF)/American Heart Association (AHA)/World Health Foundation (WHF) states that a 10-fold increase from baseline in biomarkers is recommended along with ancillary criteria, such as new pathological Q-waves, or new left bundle-branch block (LBBB) and/or imaging, or angiographic evidence of new occlusion of native vessels or grafts, new regional wall motion abnormality, or loss of viable myocardium.^[1]^

Nevertheless, the diagnosis of MI in this specific clinical setting is based on arbitrary definitions,^[1]^ because the threshold for a worsening prognosis related to an asymptomatic increase in cardiac biomarker values is still not well defined. Uncertainty remains about which is the preferred cardiac biomarker, especially with the recently developed high-sensitivity troponin assays that could be too sensitive in this scenario. Furthermore, the avoidance of cardiopulmonary bypass in off-pump CABG may lead to lower levels of biomarker release compared with on-pump procedures.^[2]^

Thus, this study aimed to quantify the release of troponin I (cTnI) and creatine kinase MB fraction (CK-MB) after CABG performed without cardiopulmonary bypass (off-pump coronary artery bypass grafting [OPCAB]) in the absence of myocardial necrosis identified by cardiac magnetic resonance (CMR) imaging with new late gadolinium enhancement (LGE) and compared them with the established values of the current universal definition of MI.

## 2 Methods

### 2.1 Population

Details of the Medicine, Angioplasty, or Surgery Study V (MASS-V) study design, protocol, patient selection, and inclusion criteria have been previously reported.^[3]^ This is a subanalysis of a trial published previously.^[4]^ Patients with angiographically documented multivessel coronary stenosis >70% by visual assessment, stable angina, preserved ventricular function, and normal baseline cardiac biomarkers were enrolled in the current study. Furthermore, they were eligible if an off-pump procedure was deemed technically feasible. All patients underwent CMR before and after the surgical procedure.

Exclusion criteria were as follows: presence of new late enhancement on CMR after the procedure, recent MI (≤6 months); signs of manifested or suspected infections or rheumatologic disease activity; chronic renal failure (creatinine level > 2.0 mg/dL); recent (≤6 months) pulmonary embolism or venous thromboembolism; patients who did not sign the consent form; and any contraindication to CMR examination, such as the presence of a pacemaker or severe claustrophobia.

### 2.2 Biochemistry

Blood samples were collected from each patient for measurement of cTnI and CK-MB mass before OPCAB and 6, 12, 24, 36, 48, and 72 h after the procedure. All the samples were centrifuged at 3000 rpm for 20 min and analyzed within 2 h after specimen collection. cTnI and CK-MB analyses were performed in an immunoassay analyzer (ADVIA Centaur, Siemens Health Care Diagnostics, Tarrytown, NY). According to the manufacturer, the lower limit of detection of cTnI using a high-sensitivity kit, the ultra kit, was 0.006 ng/mL, and the 99th percentile and MI reference limits were, respectively, 0.04 and 0.76 ng/mL. The assay precision represented by the percentage coefficient of variation (% CV) was ≤10% at 0.03 ng/mL.

The detection limit of the CK-MB mass kit (Acute Care, CK-MB assay, Siemens) was 0.18 ng/mL, and the cutoff values at the 99th percentile, established by our laboratory, were 3.8 ng/mL for women and 4.4 ng/mL for men. The CVs for CK-MB mass, as specified by the manufacturer, were 3.91% at 3.55 ng/mL for women and 3.61% at 80.16 ng/mL for men.

### 2.3 Surgical technique

Trial operators were required to perform optimum coronary revascularization in accordance with current best practices. The same team of cardiac surgeons with experience in OPCAB surgery performed the procedures in a standardized fashion. Surgical access to the heart was through a standard median sternotomy in all cases. All incisions and closure techniques were performed in the same way in all patients to limit variability among patients. The Octopus stabilizer described in detail elsewhere was used in OPCAB surgery.^[5]^ Briefly, the distal ends of the 2 suction arms of the stabilizer are placed on the beating heart on both sides of the target coronary artery. The proximal parts are fixed to the operating table. Through the application of negative pressure, the target area of the heart is sufficiently immobilized to allow the safe construction of the anastomosis of the graft with the recipient artery.

### 2.4 CMR protocol

The patients underwent scanning with a 1.5-T clinical MR scanner (Philips Healthcare, Andover, MA), and steady-state free-procession cine images were acquired in 2 long-axis (2 and 4 ventricles) views. A gadolinium-based contrast agent (Gd-DOTA marketed as Dotarem; Guerbet S.A., Paris, France) was injected intravenously (0.1 mmol/kg of body weight), and contrast-enhanced images were acquired after a 5- to 10-min delay with the use of an inversion-recovery segmented sequence. Contrast-enhanced images were acquired in long- and short-axis planes identical to the cine images. Typical voxel size was 1.6 × 2.1 × 8 mm, with a reconstruction matrix of 528 and a reconstructed voxel size of 0.6 mm. The method for acquiring and analyzing CMR was standardized in our service and was reproduced according to conventional techniques. Delayed enhancement of CMR was performed with a phase-sensitive inversion recovery sequence (repetition time 6.1, echo time 3.0 ms, voxel size 1.6 × 2.1 × 8 mm, flip angle 25°) following a 5-min time delay after the administration of 0.1 mmol/kg contrast agent (Gadoteratemeglumine Gd-DOTA, Guerbet SA). Images were acquired in 2 long-axis planes and in a short-axis stack covering the entire left ventricle. The inversion time was meticulously adjusted throughout the acquisition to obtain optimal nulling of remote normal myocardium. The slice thickness at the apex was reduced to 5 mm to avoid a partial volume effect.

### 2.5 Ethics committee approval

All patients provided written informed consent and were assigned to a treatment group. The Ethics Committee of the Heart Institute of the University of São Paulo Medical School, São Paulo, SP, Brazil approved the trial. All procedures were performed in accordance with the Declaration of Helsinki.

### 2.6 Statistical analysis

Values are expressed as mean (± standard deviation) or median (interquartile range), as appropriate. The paired-sample *t* test and the unpaired-sample *t* test were used to compare means within the study group or between subgroups. Chi-squared statistics with Fisher exact test were used for comparison of discrete variables. Continuous variables that were not distributed normally were compared with the Mann–Whitney *U* test. A probability value of <0.05 was considered statistically significant.

## 3 Results

**Figure 1:**
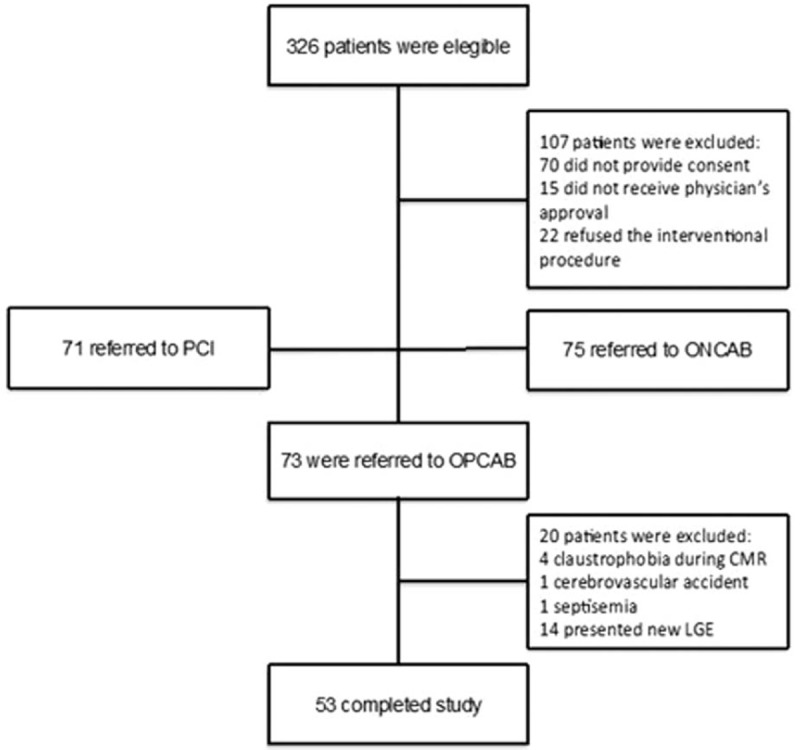
Consolidated Standards of Reporting Trials diagram. CMR = cardiac magnetic resonance, LGE = late gadolinium enhancement, ONCAP = on-pump coronary artery bypass, OPCAB = off-pump coronary artery bypass, PCI = percutaneous coronary intervention.

Between March 2012 and March 2014, 326 prospective consecutive patients from the Department of Atherosclerosis at the Heart Institute (InCor) of the University of São Paulo Medical School were evaluated. Of these, 107 were excluded for various reasons; 71 were referred for percutaneous coronary intervention (PCI) and 75 to on-pump CABG. From the 73 patients enrolled in this study, 20 were excluded and 53 completed the study protocol. The main reasons for the exclusions are shown in Fig. 1.

The baseline characteristics were summarized in Table 1. The mean age was 63 ± 10 years, and 37 (70%) were males. Also, 51% of patients were diagnosed with type 2 diabetes mellitus, and 40% of patients had a history of MI. Twelve patients (7%) continued smoking during the study period. Furthermore, the average SYNTAX score was 20 ± 7, and the mean ejection fraction assessed by CMR was 69 ± 9.

**Table 1:**
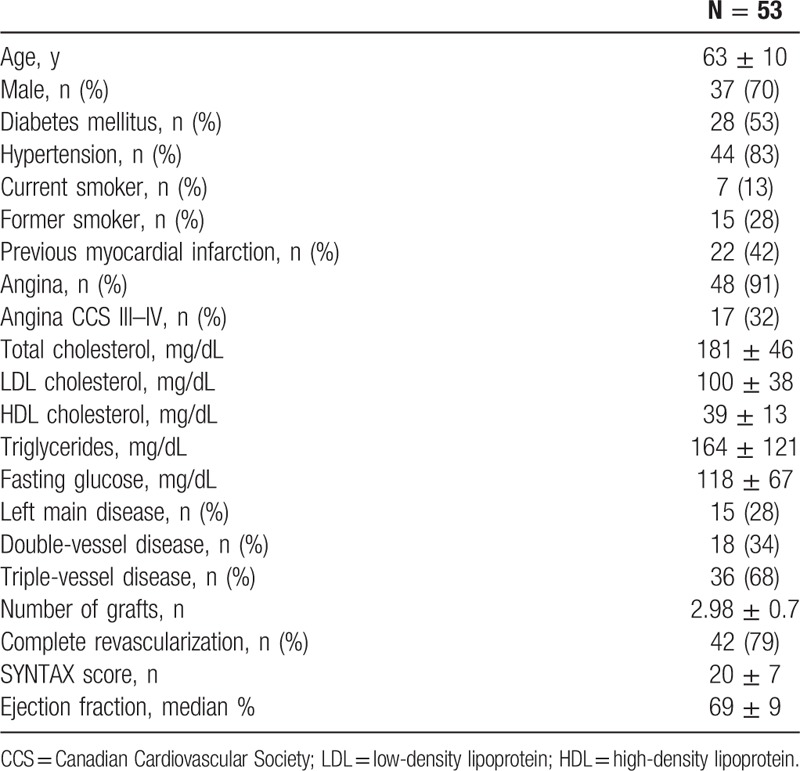
Characteristics, demographic profile, and clinical profile.

No patient had evidence of new “Q” wave pathology or new LBBB activity on the electrocardiogram performed after CABG.

### 3.1 Biomarkers

The median value of the peak cTn was 2.0 ng/mL, which corresponds to a 50-fold increase from the 99th percentile. All patients had cTnI values below the 99th percentile before the procedure.

Of these patients, 5 (9%) had elevation between the 99th percentile and the threshold of 10 times the 99th percentile. The 48 (91%) remaining patients had elevation above the threshold of 10 times the 99th percentile. No patient had a cTn value below the 99th percentile after surgery.

Regarding the biomarker CK-MB mass, the median peak value was 14.6 ng/mL for females and 12.7 ng/mL for males. All patients had CK-MB values below the 99th percentile before the procedure.

**Figure 2:**
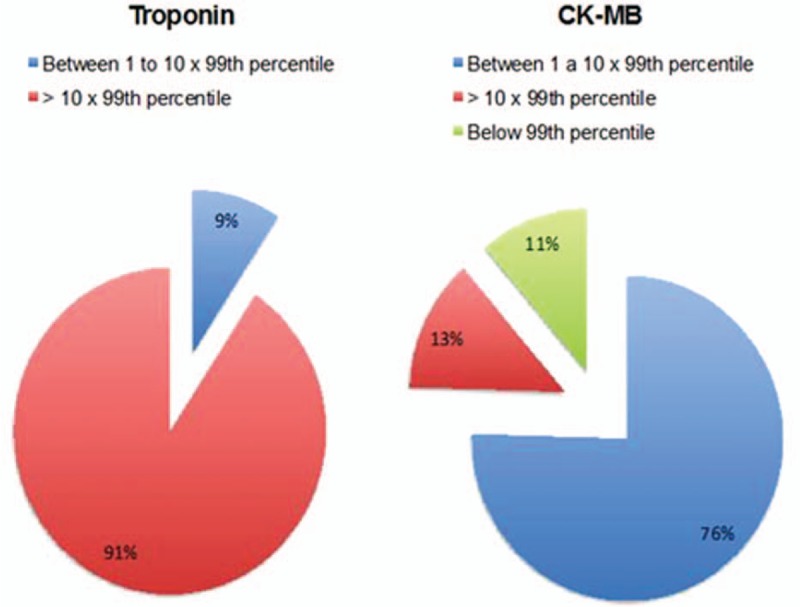
Proportions of the release of cardiac biomarkers.

Additionally, we noted that 40 (76%) patients had an elevation between the 99th percentile and the threshold of 10 times the 99th percentile, 7 (13%) had peak values higher than 10 times the 99th percentile, and 6 (11%) had no elevation in this biomarker (Fig. 2).

**Figure 3:**
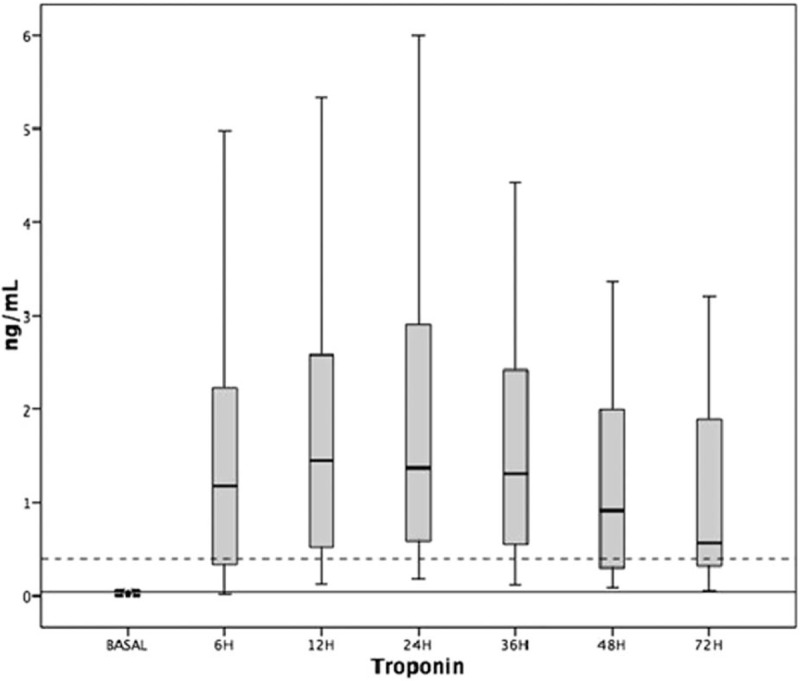
Distribution of cTn release for each time of evaluation. Continuous line shows the value of the 99th percentile. Dotted line shows the value of 10 times the 99th percentile.

The pattern of cTn elevation in each moment of evaluation after surgery is described in the chart shown in Fig. 3.

The comparisons of the levels of cTn in the different periods after the procedure showed a statistically significant difference, *P* < 0.01 in all groups.

**Figure 4:**
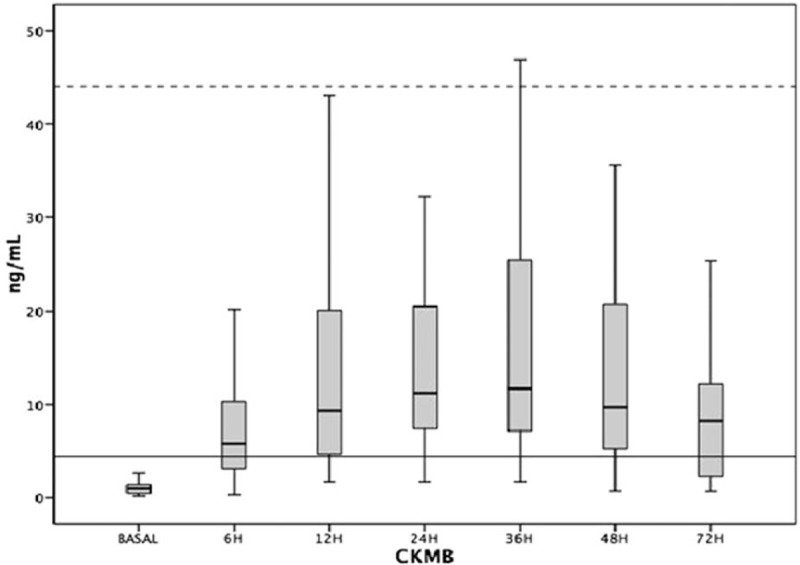
Distribution of CK-MB release at each time of evaluation in male patients. Continuous line shows the value of the 99th percentile. Dotted line shows the value of 10 times the 99th percentile. CK-MB = creatine kinase MB fraction.

**Figure 5:**
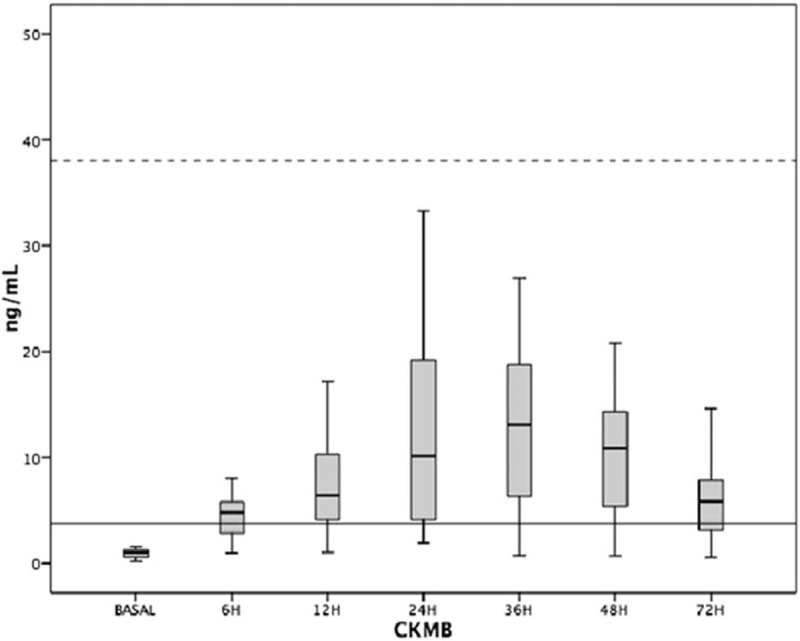
Distribution of CK-MB release at each time of evaluation in female patients. Continuous line shows the value of the 99th percentile. Dotted line shows the value of 10 times the 99th percentile. CK-MB = creatine kinase MB fraction.

The pattern of CK-MB elevation in each moment of evaluation after surgery for males and females is described in the chart shown in, respectively, Figs. 4 and 5.

The comparisons of CK-MB values in the different periods after the procedure showed statistically significant differences, *P* < 0.01 in both groups.

## 4 Discussion

In this study, based on the cutoffs recommended by the current guidelines, we found marked differences in the results, depending on which biomarker was analyzed. The elevation of cTnI was above the 99th percentile in all patients after surgery, with the majority exceeding 10 times the 99th percentile value reaching up to 50 times the cutoff. On the other hand, the release of CK-MB was predominantly below the 10-fold 99th percentile threshold. Thus, cTn falsely indicated MI in the majority of patients, while CK-MB elevation was consistent with myocardial injury related to the cardiac procedure. Of note, both patterns occurred regardless of the absence of new LGE in CMR performed after OPCAB. Moreover, all patients experienced good recovery after cardiac revascularization.

Numerous reasons can lead to periprocedural myocardial injury with release of cardiac biomarkers after CABG. These include direct myocardial trauma from suture placement or manipulation of the heart, inadequate intraoperative cardiac protection, microvascular events related to reperfusion, myocardial injury induced by oxygen free radical generation, or failure to reperfuse areas of the myocardium that are not subtended by graft table vessels. It should be noted that biomarker elevations after CABG could be secondary to a variety of factors, including underlying pathology, surgical technique, and perioperative complications, and may not be indicative of a vascular event.^[1,6]^

The current ESC/ACCF/AHA/WHF statement advocates the use of cTn assay for detection and quantification of type 5 MI. Nevertheless, the current threshold was primarily based on studies that reported a significant correlation of CK-MB with adverse events, such as death, shock, and prolonged length of stay in the intensive care unit.^[7]^ Klatte et al reported that CK-MB was an independent predictor of 6-month mortality (odds ratio [OR] 1.90 [1.04–3.48], 1.97 [0.94–4.14], and 4.78 [2.37–9.64] for 5–10, 10–20, and >20 times the upper limit of normal [ULN], respectively). Moreover, the analysis of the consecutive area under the receiver operating characteristics curve suggested that the optimal cutoff point ranged from 5 to 10 times the ULN. Higher cutoff values (>10 times the ULN) were associated with higher specificity and negative predictive values, yet suffered from lower sensitivity.^[8]^ Domanski et al in a meta-analysis demonstrated increased cardiovascular mortality within 30 days in patients with CK-MB elevation >5 times the 99th percentile and with an elevation of cTn above 40 times the 99th percentile.^[6]^ Hence, these studies assessed CK-MB levels and associated its elevations with cardiac events. The extrapolation from these results of cTn thresholds and diagnosis of periprocedural MI should be made carefully. The main drawback of these trials is that the group of individuals who reach the 5 times threshold of cardiac biomarkers is essentially different. There were those who had an isolated rise in biomarkers among those with real myocardial necrosis and consequently impaired prognosis. Therefore, the prognosis may be driven by the extent of necrosis, regardless of the cause of injury.

Furthermore, the transition from CK-MB results to cTn results was made prior to the thorough understanding of the prognostic implications of cTn elevation after CABG.^[9]^ The criterion based on the threshold of 10-fold upper reference limit for cTn alone is clearly too sensitive, but higher thresholds appear to be more accurate.^[4]^ Pegg et al assessed the utility of biomarkers cTnI and CK-MB after CABG associated with results of CMR imaging. cTn elevation was observed above the value of 10 times the 99th percentile in all patients. The median value of cTnI peaks was 4.7 ng/mL in patients without further delayed enhancement in CMR after the procedure, which corresponds to twice the value found in our study. However, the median value of CK-MB peak was 12.4 ng/mL, a similar value to that found in our study. Of note, this study applied the same immunoassay (Siemens, ADVIA Centaur) as that used in our trial.^[10]^ However, differently from the present study, the authors included in the analysis 2 different operative techniques and patients with impaired ventricular function. It has been shown that on-pump and off-pump procedures are associated with distinct patterns of cardiac biomarker release.^[4]^

The present study confirms that cTn levels are usually elevated after off-pump CABG. Impaired outcome has been reported for cTn values that were elevated to the highest quartile of quintile of the measurements. A cTnT threshold (1.58 ng/mL) was chosen by Januzzi et al, who reported a correlation with in-hospital mortality (OR 31.0 [95% confidence interval, CI, 3.67–263.1]) and death/MI (OR 60.1 [95% CI 7.34–492.1]). The same authors have demonstrated that cardiac cTnT levels in the upper quintile were significant independent predictors of in-hospital complications. In this cohort, both cTnT levels taken immediately postoperatively and those assayed between 18 to 24 h after surgery were independently predictive of a complicated in-hospital course.^[11]^ They also showed that cTnT levels were higher in this respect than measures of CK-MB. The same authors have also reported the 1-year outcome of 136 patients who underwent isolated CABG. During this time, 7 (5%) patients died. They found that median cTnT levels were higher among those patients who died, and that a cTnT level in the highest quintile at 18 to 24 h was the strongest independent predictor of mortality (OR 5.45, 95% CI 4.5–232.5, *P* < 0.01).^[12]^ Similarly, Croal et al assessed the prognostic importance of cTnI in 1365 patients undergoing cardiac surgery who were followed up at 30 days, 1 year, and 3 years after surgery. Their study confirmed that cTnI levels are frequently elevated, and patients with cTnI levels in the highest quartile are at higher risk.^[13]^ Again, all these studies assessed cTn levels and correlated them to cardiac events. They did not evaluate MI and thus, regarding the proposed MI definition, outcomes from these studies should be interpreted with caution.

Unlike CK-MB, the interpretation of cTn varies depending on whether cTnT or cTnI is used and which of the variety of available cTnI methods is used. Moreover, the lack of standardizations of such assays adds complexity to the diagnosis. The thresholds for cTn are no longer comparable to the historic values, because of reductions to the lower limit of detection seen with new-generation cTn assays that have altered the ability to detect myocyte necrosis. Finally, the type of operation is a significant confounder when one interprets postoperative biomarker levels, because more complex surgeries are associated with greater elevations. This should be taken into account when values are interpreted. The greater elevations are likely associated with several features, including length of time on bypass and myocardial damage due to the surgery itself.

Among the noninvasive imaging methods recommended for detection of MI after postoperative myocardial revascularization, CMR imaging was used in our study to exclude from our analysis those patients with new detectable areas of necrosis after surgery. CMR has been used for assessment of coronary hypoperfusion extension and myocardial necrosis. Kim et al reported sensitivity to myocardial necrosis detection of up to 99% in the acute phase of MI.^[14]^ In addition, Wu et al identified focal images of delayed enhancement by gadolinium in the CMR after acute MI, with detection areas up to 0.16 g.^[15]^ However, CMR studies have reported that cTnI has a low discriminatory power for detecting MI after coronary artery bypass procedures. Lim et al reported excessive sensitivity of cTnI compared to CK-MB in patients after PCI, even in the absence of new delayed enhancement in the CMR.^[16]^ Van Gaal et al conducted a study that compared the rise in cTn and the new onset of delayed enhancement in the CMR after surgical and percutaneous revascularization. It was observed that all patients had elevated cTn, even those with no evidence of new LGE on CMR after surgery.^[17]^ In addition, the average SYNTAX score value was similar to the value obtained in our study and showed no statistically significant difference between the groups with or without new delayed cardiac enhancement after the procedure. Thus, CMR studies have demonstrated that usual cTnI elevations are poor for detecting type 5 MI. Although light elevations of cTnI may be due to myonecrosis not detectable by CMR, the prognostic significance of such minor elevations has not been associated with a worse prognosis. Moreover, even using CMR that is the most sensitive imaging technique so far to evaluate myocardial fibrosis, the regular biomarker elevation did not reveal signs of MI.

In conclusion, assuming that biomarkers of myocardial necrosis have limited diagnostic accuracy in myocardial injury, the challenges faced for the establishment of definitive values for the diagnosis of myocardial damage include new cutoff values for cTnI. Troponin did not indicate injury and MI by the excessive sensitivity demonstrated and by the arbitrary limit of 10-fold the 99th percentile threshold. cTn may still be valuable in this context, but its thresholds should be carefully reviewed in future studies. When the current definition of MI after OPCAB was used, CK-MB had greater accuracy and should be reviewed as a marker of choice.
